# Diagnostic value of an increase in central venous pressure during SBT for prediction of weaning failure in mechanically ventilated patients: A cross‐sectional study

**DOI:** 10.1002/hsr2.1204

**Published:** 2023-04-13

**Authors:** Ali Akbar Ghamari, Keivan Amini, Amin Daei Sorkhabi, Aila Sarkesh, Seyed Hadi Saghaleini, Roghayeh Asghari, Mansour Rezayi, Ata Mahmoodpoor

**Affiliations:** ^1^ Intensive Care Unit, Imam Reza Hospital Tabriz University of Medical Sciences Tabriz Iran; ^2^ Student Research Committee Tabriz University of Medical Sciences Tabriz Iran

**Keywords:** central venous pressure, extubation, mechanical ventilation, spontaneous breathing trial, weaning

## Abstract

**Background:**

Timely and successful extubation is an essential step forward in clinical practice to minimize complications of mechanical ventilation and unsuccessful weaning processes. Thus, research into predictive factors of weaning outcome to optimize spontaneous breathing trial (SBT) precision before extubation is critical in intensive care practices. In this study, we aimed to investigate the predictive factors of the weaning outcome before and during SBT in mechanically ventilated patients.

**Methods:**

In this cross‐sectional study, 159 mechanically ventilated patients who were eligible for SBT were enrolled. Of these patients, 140 had successful extubation, whereas the remainder failed. Each patient's PaCO_2_ and PaO_2_ levels, respiratory rate (RR), SpO_2_, mean arterial pressure (MAP), heart rate (HR), and central venous pressure (CVP) values at the start of SBT, 3 min later, and at the end of SBT were measured. These values, along with the patients' clinical characteristics, were then investigated to determine if there was any correlation between these variables and the weaning outcome.

**Results:**

Our analysis revealed that increase in CVP, independent of hemoglobin (Hb) concentration, PaO_2_, SpO_2_, duration of mechanical ventilation (MV), length of intensive care unit (ICU) stay, and SBT process, as well as underlying disease, was positively correlated with extubation/weaning failure. While age, gender, vital signs (MAP, RR, and HR), sequential organ failure assessment (SOFA), and acute physiology and chronic health evaluation (APACHE) scores had no significant correlation with patients' extubation outcomes.

**Conclusion:**

According to our findings, integrating CVP assessment into SBT besides routine indices measurement and monitoring can be considered for the prediction of weaning outcome in critically ill mechanically ventilated patients.

## 1 INTRODUCTION

In routine clinical practice, weaning is accomplished using a standardized process known as spontaneous breathing trial (SBT), which involves a real‐life test of breathing without mechanical ventilation performed before extubation to validate the patient's capacity to breathe without support. SBT estimates the risk of unsuccessful weaning by analyzing the physiological responses to the respiratory effort induced by the cessation of positive pressure breathing during a 30–120‐min period.^1^, ^2^ Even though SBT can cause complications for some patients, such as increased cardiovascular and physical stress, impaired cardiac function, arrhythmia, or ischemia, so switching from mechanical to spontaneous ventilation can reduce left ventricular function by raising preload and afterload and uncovering latent left ventricular failure.^3^ Additionally, as evidenced by recent studies, SBT failure might lead to greater muscular effort and fatigue, indicating that SBT can pose risks and complications for the patient.^4^ As a result, it is essential to investigate potential factors that are not included in routine SBT and identify individuals at high risk of unsuccessful extubation before or even during the early minutes of SBT that have not yet posed complications to the patient.^5^ Unsuccessful extubation with emergency re‐intubation is a serious consequence of the failed weaning process, which leads to enhanced mortality.^6^

Cardiovascular dysfunction is being recognized as a frequent cause of weaning failure that can be successfully treated. Clinically, it may be challenging to diagnose and distinguish cardiovascular pathophysiology as a primary risk factor or contributor to weaning failure from noncardiac causes, demanding high suspicion for its early diagnosis and intervention.^7^ Switching a patient from positive pressure ventilation to spontaneous breathing restores negative inspiratory intrathoracic pressures, increasing venous return (left ventricular preload), central blood volume, and left ventricular afterload. It is best addressed before weaning tests since it is associated with a poor prognosis for extubation/weaning.^8^ A recent study found that evaluating volume status and response to the volume before SBT is associated with a higher risk of pulmonary edema induced by weaning,^9^ thus, monitoring volume status before isolation is not practicable in all patients. Furthermore, in cases of unsuccessful extubation with the cardiovascular origin, a simultaneous rise in pulmonary artery occlusion pressure and central venous pressure (CVP) has been demonstrated.^10^

Given the above reflection, we aimed to investigate the predictive factors of the weaning outcome before and during SBT in mechanically ventilated patients, with the hypothesis that an early rise in CVP during SBT might be a signal of heart failure and unsuccessful extubation/weaning.

## 2 MATERIALS AND METHODS

### 2.1 Study design

This cross‐sectional study was carried out on critically ill patients undergoing mechanical ventilation who had been hospitalized for more than 48 h in the ICUs of Shohada and Imam Reza hospitals (two university‐affiliated hospitals in the northwest of Iran). The protocol of this study was reviewed and approved by the Ethics Committee of Tabriz University of Medical Sciences (IR.TBZMED.REC.1399.364), and all participants or their next of kins were fully informed of the information and provided written consent.

### 2.2 Study subjects

We recruited 159 patients based on the following inclusion and exclusion criteria. The main criterion for inclusion in this study was that the patient met the clinical criteria for weaning or SBT. Additional inclusion criteria were stable hemodynamics, assisted mechanical ventilation (FiO_2_ ≤ 50%, PaO_2_/FiO_2_ > 200, positive end‐expiratory pressure (PEEP) <6 cmH_2_O during 4–6 h before SBT), and an adequate cough reflex, arterial pH ≥ 7.35, the presence of a CVP catheter, and hemoglobin (Hb) above 10 g/dL. Conversely, age less than 18 years, persistent neurological impairment with a low level of consciousness, uncontrolled infection/sepsis, unstable hemodynamics, medium to high‐dose vasopressor‐dependence, need for norepinephrine more than 0.3 μg/kg, presence of tracheostomy tube, and a do‐not‐resuscitate (DNR) order were all exclusion criteria.

### 2.3 Weaning trial

Patients who were eligible for SBT were ventilated with pressure support ventilation (PSV) and PEEP before the procedure, as per the department's protocol (PSV = 6–8 cmH_2_O and PEEP: 5–6 cmH_2_O). SBT was performed with a T tube connected to oxygen while the patient was supine and at 30°, with no change in the patient's position during SBT. The co‐executor of the protocol determined the duration of the T tube at 120 min, and if tolerated, the patient was prepared for extubation. If the patient exhibited signs of intolerance during this period, SBT was discontinued and the patient was returned to PSV. Clinical criteria used to determine whether SBT was successful were a respiratory rate (RR) <35, less than a 20% rise over baseline heart rate (HR), oxygen saturation (SpO_2_) >90%, any change less than 25% in blood pressure, and the absence of signs of increased respiratory work or respiratory distress (contraction of lateral muscles, paradoxical or asynchronous chest movements, intercostal contractions, diaphoresis, nasal flaring, and agitation). The decision to extubate a patient after successful SBT and optimal consciousness was made based on the patient's clinical status. If the patient's condition remained stable after extubation for 48 h, the extubation was considered successful, and clinical follow‐up was discontinued after 48 h. Conversely, if extubation/weaning failed within 48 h, patients were re‐intubated, and the reasons for the need for re‐intubation, the duration between the patient's spontaneous breathing and the need for mechanical ventilation, PaCO_2_ and PaO_2_, RR, SpO_2_, mean arterial pressure (MAP), HR, and CVP variables were evaluated and recorded during re‐intubation and after intubation. Also, oxygen therapy was performed with conventional O_2_ therapy. After extubation, all patients received O_2_ via face mask (simple or with a reservoir bag, based on their oxygenation). If the condition worsened, they would receive noninvasive ventilation or would get intubated and undergo mechanical ventilation, depending on their condition.

### 2.4 CVP test

Among other factors, CVP was measured at the initiation of SBT, 3 min later, and at the end. CVP was measured by a nurse who was blinded to the study, and the measurement was done by one nurse in each center to minimize interobserver variability. The CVP measurement method was to first decrease PEEP to 0, and then CVP was measured at the end of exhalation using a CV line inserted into the internal jugular or subclavian vein in the fourth intercostal space at the midaxillary line.

### 2.5 Statistical analysis

The normal distribution of the data was evaluated using the Kolmogorov–Smirnov test. Parametric variables were expressed as mean ± SD (standard deviation) and nonparametric variables were expressed as a median. As well, nominal variables were expressed as percentages. To compare parametric and nonparametric variables, the Mann–Whitney *U* and *T*‐tests were employed, and for categorical variables, the *χ* ^2^ test or Fisher's exact test was applied. Also, a multivariate logistic regression test was performed 3 min after the start of SBT to evaluate the predictive value of variables including, body mass index (BMI), length of intensive care unit (ICU) stay, duration of mechanical ventilation, Hb concentration, acute physiology and chronic health evaluation (APACHE) score, sequential organ failure assessment (SOFA) score, HR, RR, MAP, SpO_2_, and CVP. Statistical analyses were performed using the statistical software package SPSS version 23.0 (SPSS Inc., Chicago, IL, USA). The *p* < 0.05 was considered statistically significant.

## 3 RESULTS

A total of 159 patients were recruited, comprising 110 (69.2%) men and 49 (30.8%) women. Patients ranged in age from 28 to 78, with a mean age of 62. The baseline characteristics of patients are presented in Table 1. Extubation was successful in 140 (88.1%) of the 159 patients, whereas it failed in 19 (11.9%), with re‐intubation undertaken in 17 (89.5%) and 2 (10.5%) patients due to respiratory failure and loss of consciousness, respectively. SBT, however, was successful in 131 (82.4%) patients and failed in 28 (17.6%).

**Table 1:** Clinical findings of the studied population.

Characteristics	Successful extubation group (mean ± SD) *N* = 140	Extubation failure group (mean ± SD) *N* = 19	*p* Value
Age (years)	61.87 ± 11.03	62.89 ± 7.49	0.69
Sex (male/female)	99/41	11/8	0.256
BMI (kg/m^2^)	26.65 ± 2.92	26.47 ± 3.42	0.81
Hb (g/dL)	11.59 ± 1.1	10.95 ± 1.22	**0.02**
APACHE score	21.17 ± 5.42	20.47 ± 4.32	0.59
Baseline SOFA score	10.14 ± 2.39	10.42 ± 2.83	0.64
SOFA score at the day of extubation	5.71 ± 1.52	6.26 ± 1.28	0.13
ICU length of stay (days)	12.48 ± 4.25	15.42 ± 5.14	**0.007**
Duration of mechanical ventilation (days)	8.84 ± 3.77	12.05 ± 4.4	**0.001**
CVP during mechanical ventilation (mmHg)	10.63 ± 1.53	10.42 ± 1.42	0.56
Re‐intubation time after extubation failure (h)	—	31.36 ± 11.64	—
SBT duration (min)	107.6 ± 10.02	113.68 ± 10.52	**0.01**
***Diagnosed disease % (number)***			
Sepsis	6.4 (9)	10.5 (2)	0.734
Respiratory disease	25 (35)	36.8 (7)	
Cardiovascular disease	4.3 (6)	0 (0)	
Gastrointestinal disease	10 (14)	10.5 (2)	
Central nervous system disease	24.3 (34)	26.3 (5)	
Post‐surgery	3.6 (5)	0 (0)	
Trauma	26.4 (37)	15.8 (3)	
***Underlying disease % (number)***			
None	25 (35)	31.6 (6)	**0.02**
Hypertension	22.9 (32)	21.1 (4)	
Diabetes mellitus	17.9 (25)	21.1 (4)	
Heart failure	5.7 (8)	0 (0)	
Ischemic heart disease	14.3 (20)	0 (0)	
Hypertension/diabetes mellitus	1.4 (2)	5.3 (1)	
Hypertension/heart failure	1.4 (2)	0 (0)	
Hypertension/ischemic heart disease	5 (7)	5.3 (1)	
Diabetes mellitus/heart failure	0 (0)	10.5 (2)	
Diabetes mellitus/ischemic heart disease	1.4 (2)	0 (0)	
Others (COPD, CVA, obesity, AKI, and HLP)	5 (7)	5.3 (1)	

*Note*: *p* < 0.05 was considered statistically significant.

Abbreviations: AKI, acute kidney injury; APACHE, physiology and chronic health evaluation; BMI, body mass index; COPD, chronic obstructive pulmonary disease; CVA, cerebrovascular accident; CVP, central venous pressure; Hb, hemoglobin; ICU, intensive care unit; SOFA, sequential organ failure assessment.

According to our analysis, patients' gender (*p* = 0.256), age (*p* = 0.69), and BMI (*p* = 0.81) had no significant impact on extubation outcome. However, in contrast to diagnosed disease (*p* = 0.734), patients' underlying comorbidities (*p* = 0.02) had a significant impact on the extubation outcome. Also, compared to patients with successful extubation, ICU length of stay (*p* = 0.007), duration of mechanical ventilation (*p* = 0.001), and SBT duration (*p* = 0.01) were significantly greater in patients with extubation failure. On the other hand, the Hb concentration (*p* = 0.02) was significantly higher in successful extubation patients compared to extubation failure patients. While, there was no significant difference found in APACHE score (*p* = 0.59), SOFA score at baseline (*p* = 0.64) and extubation (*p* = 0.13), and CVP during mechanical ventilation (*p* = 0.56) among patients with successful and failed extubation.

During SBT, there was no significant difference in HR, MAP, RR, or PaCO_2_ between patients with successful and failed extubation. However, at the end of SBT, SpO_2_ (*p* < 0.0001) and PaO_2_ (*p* = 0.002) were substantially higher in patients who were successfully extubated compared to their unsuccessful counterparts, but there was no significant difference in their values at baseline and 3 min after SBT initiation. Likewise, in extubation failure patients, the CVP was significantly higher 3 min after SBT started (*p* < 0.0001) and at the end of SBT (*p* < 0.0001) compared to patients with successful extubation (Table 2).

**Table 2:** The correlation between extubation outcome and vital signs, respiratory parameters, and CVP of the mechanically ventilated patients.

Variable		Successful extubation group (mean ± SD) *N* = 140	Extubation failure group (mean ± SD) *N* = 19	*p* Value
HR (beats/min)	Base	70.3 ± 8.63	73.84 ± 73.84	0.09
	3 min	72.03 ± 8.47	74.31 ± 7.34	0.26
	End of SBT	75.59 ± 7.9	76.05 ± 9.08	0.81
	Re‐intubation	–	84.1 ± 3.75	–
	Post re‐intubation	–	82.89 ± 13.85	–
MAP (mmHg)	Base	72.26 ± 4.5	71.57 ± 3.27	0.52
	3 min	72.4 ± 34.65	69.31 ± 17.3	0.08
	End of SBT	74.87 ± 5.62	73.16 ± 5.84	0.21
	Re‐intubation	–	74.73 ± 7.25	–
	Post re‐intubation	–	68.31 ± 7.15	–
SpO_2_ (mmHg)	Base	94.33 ± 1.34	94.26 ± 1.14	0.82
	3 min	93.82 ± 1.66	93.57 ± 1.34	0.53
	End of SBT	94.24 ± 1.5	92.78 ± 1.68	**<0.0001**
	Re‐intubation	–	91.31 ± 1.97	–
	Post re‐intubation	–	93.47 ± 1.38	–
RR (breath/minute)	Base	22.45 ± 2.88	21.73 ± 2.82	0.3
	3 min	23 ± 3.34	21.94 ± 2.24	0.18
	End of SBT	23.98 ± 3.14	23.84 ± 5.55	0.86
	Re‐intubation	–	27.52 ± 8.51	–
	Post re‐intubation	–	24.84 ± 6.44	–
CVP (mmHg)	Base	8.72 ± 1.35	8.57 ± 1.38	0.65
	3 min	8.32 ± 1.29	11.15 ± 1.86	**<0.0001**
	End of SBT	8.21 ± 1.3	10.05 ± 1.68	**<0.0001**
	Re‐intubation	–	10.26 ± 1.96	–
	Post re‐intubation	–	9.52 ± 1.57	–
PaO_2_ (mmHg)	Base	65.37 ± 4.6	66.89 ± 3.57	0.16
	3 min	65.48 ± 5.01	67.42 ± 3.94	0.1
	End of SBT	68.02 ± 5.46	63.84 ± 4.19	**0.002**
	Re‐intubation	–	58.57 ± 5.21	–
	Post re‐intubation	–	63.47 ± 4.8	–
PaCO_2_ (mmHg)	Base	43.8 ± 4.28	42.52 ± 4.03	0.22
	3 min	43.44 ± 4.7	41.52 ± 3.9	0.09
	End of SBT	44.3 ± 6.17	43.26 ± 3.91	0.47
	Re‐intubation	–	47 ± 12.18	–
	Post re‐intubation	–	46.36 ± 1.8	–

*Note*: *p* < 0.05 was considered statistically significant.

Abbreviations: CVP, central venous pressure; HR, heart rate; MAP, mean arterial pressure; RR, respiratory rate; SBT, spontaneous breathing trial.

## 4 DISCUSSION

The success or failure of an SBT is determined by objective and subjective criteria, as well as whether it leads to successful extubation or necessitates re‐intubation. Given the fact that these criteria and instructions do not include the CVP, our study demonstrated that elevated CVP during SBT is an independent indicator that is positively associated with weaning failure.

CVP is defined as the interaction between the cardiac and venous return curves, which may be influenced by discontinuing positive pressure mechanical ventilation. The normal CVP response during SBT typically declines in patients with normal cardiac function and without fluid overload or strong inspiratory efforts.^11^ As a result, the initial increase in CVP may be interpreted as abnormal in the context of SBT. CVP values are significantly influenced by strong respiratory efforts that result in fluctuations in negative pleural pressure.^12^ However, since the CVP assessment was accomplished only 3 min after the end of positive pressure ventilation and SBT, it is unlikely that this factor impacted the CVP values clinically. In this way, Dubo and colleagues demonstrated that CVP increased significantly in the early minutes of SBT, but they could not clarify the underlying reasons for the absence of this pattern at the end of SBT. Nonetheless, they highlighted the potential significance of increased CVP as a potent but transient early warning sign that is best acknowledged after the commencement of SBT.^13^ Likewise, according to a study by Cao and colleagues, weaning failure is associated with CVP elevation before extubation.^7^, ^14^ Consistently, our research confirmed these findings and showed that there was no correlation between the rise in CVP level after extubation and weaning outcome.

According to previous research, one of the most common reasons for extubation failure is cardiac dysfunction, which includes obvious or even occult systolic or diastolic dysfunction, as well as coronary heart disease, arrhythmias, and other cardiac complications that compromise the likelihood of successful weaning.^9^, ^10^, ^15^ Moreover, psychological stress and possible hypoxia during weaning/extubation may lead to sympathetic activation. Many critically ill patients have undiagnosed or subclinical cardiovascular diseases and insufficient cardiac reserve may result in subsequent respiratory insufficiency and weaning failure.^16^ Detrimental loading conditions for the right heart can develop after weaning and shifting the intrathoracic pressure from positive to negative pressure. This can lead to a sharp rise in venous return and a simultaneous increase in left ventricular afterload, which is accompanied by an increase in adrenergic tone during SBT.^11^, ^15^ In the context of fluid resuscitation, Weil et al.^17^ suggested that CVP be employed as a measure of venous return intolerance. Also, in a preliminary study, Lemaire et al. found that pulmonary edema occurred immediately after initiation of spontaneous breathing if CVP increased by 12 mmHg.^9^ In another study, Dres et al.^18^ found that patients who failed SBT attributable to cardiac dysfunction had a mean rise of 5 mmHg in CVP during SBT, whereas patients with successful weaning had a CVP increase of just 1 mmHg. It is difficult to evaluate cardiac dysfunction during SBT due to the need for echocardiography in patients; one reason for this is the patient's semi‐sitting position during SBT and occasionally rapid breathing, as well as constraints in available facilities. Nonetheless, CVP is a significant predictive factor that is currently not included among the conventional SBT evaluation criteria.

However, a rise in CVP should only be considered a warning sign since it does not provide diagnostic clues without further assessment.^13^ Rousti et al.^19^ demonstrated that weaning failure can be prevented and even reversed in selected individuals by assessing cardiac function and implementing drainage interventions including diuretics and vasodilators. As a result, an abnormal rise in CVP during SBT might signal the need for a rapid assessment of cardiac function in patients and contribute to a more appropriate allocation of limited resources such as echocardiography to patients.

Hb concentration was found to be positively correlated with weaning success in patients with challenging weaning in a large retrospective study by Lai et al.^20^ and lower Hb concentration was attributed to extubation failure in a smaller cohort study.^21^ Similarly, a prospective case–control study by Georgakas et al.^22^ showed that Hb concentration was positively associated with weaning outcome, which was consistent with our findings. The oxygen‐carrying capacity of the blood is directly related to the level of Hb, and in normal healthy people, 15 g/dL of Hb carries approximately 21 mL of oxygen per 100 mL of blood.^23^, ^24^ In this way, a decline in Hb levels leads to a decrease in oxygen‐carrying capacity. Since Hb saturation is commonly abnormal in patients with respiratory failure, this consequence is more prominent.^25^ Therefore, the decrease in oxygen‐carrying capacity is expected to disrupt the aerobic metabolism of respiratory muscles, culminating in breathing inefficiency and weaning failure.

Also, SpO_2_ was identified as an independent predictive factor for weaning success. This finding is consistent with previous studies that an integrated index that includes SpO_2_ has higher diagnostic accuracy than other conventional indices in predicting weaning outcomes,^24^ and a higher SpO_2_ level is positively correlated with SBT success.^22^ As arterial oxygen content is low, the blood‐to‐mitochondria oxygen diffusion gradient diminishes more rapidly, potentially driving respiratory muscles toward primary anaerobic metabolism and resulting in breathing inefficiency and weaning failure.^23^ Several research, notably the one by Teixeira and colleagues have indicated that PaO_2_ at the initiation and end of SBT cannot be distinguished between two groups with successful and failed weaning.^22^, ^26^, ^27^ In contrast, we found that the PaO_2_ level at the end of SBT was a factor associated with extubation success, but there was no correlation at baseline or 3 min after the initiation of SBT. Most patients undergoing weaning have substantial gas exchange abnormalities during the weaning process and may become hypoxemic, hypercapnic, or both during SBT.^28^ Goodgame et al.^29^ discovered that SpO_2_ was well correlated with PaO_2_, as predicted by the standard oxygen‐hemoglobin dissociation curve in a population of undifferentiated critically ill patients, confirming the coordination of SpO_2_ and PaO_2_ at the end of SPT. The decrease in PaO_2_ is triggered by the lungs' failure to supply adequate oxygen to the blood, and it has several explanations, including hypoventilation, impaired alveolar diffusion, and pulmonary shunt, which occurs when the heart fails to sufficiently pump blood.^30^ Nonetheless, the association between PaO_2_ and this finding is novel, and the diagnostic accuracy of PaO_2_ as a single indicator should be investigated in future investigations as part of the effective combination of indicators in the weaning outcome.

Tanios and colleagues previously demonstrated that using predictive cues while deciding whether to perform SBT delays extubation.^31^ However, determining the optimal timing for extubation is essential. Early extubation is linked to an increased risk of re‐intubation, a higher requirement for tracheostomy, a longer ICU stay, and a higher mortality rate.^32^ Late extubation, on the other hand, raises the risk of pneumonia, ICU stay, and mortality.^2^, ^33^ In this respect, Teixeira et al.^26^ found that the length of stay in the ICU and the duration of mechanical ventilation were significantly longer in the group that failed extubation than in the group that succeeded. According to retrospective research by Liang et al.^34^ the duration of mechanical ventilation and length of stay in the ICU were significantly longer in the weaning failure group than in the successful group. The results of the current study also indicated the positive correlation between the duration of mechanical ventilation and the length of stay in the ICU and weaning failure.

Based on strong evidence, the task force recommended in 2001 that SBT last at least 30 min and no more than 120 min.^35^ This suggests that we should wait at least 30 min before assessing SBT tolerance, but not more than 120 min if SBT tolerance is uncertain. Before deciding to continue SBT, the first few minutes should be carefully reviewed. There is evidence that respiratory muscle fatigue is detrimental if it develops early with ineffective SBTs.^36^ Our study was the first to demonstrate that the duration of the SBT process had a positive correlation with extubation failure.

Despite its strengths, our research had some limitations. Due to resource constraints and difficulties, we were unable to evaluate patients' cardiac function during SBT, which weakened the inference of causality in this study. Also, due to a lack of follow‐up after extubation failure, we were unable to provide information regarding the following SBTs of patients with extubation failure. Further, CVP measurements could be subject to interobserver variability, although we tried to minimize this risk by using a standardized method and measurements by a single nurse at each center who was blinded to the study. Additionally, while this study was conducted on general ICU patients, the study population included a relatively small number of patients with chronic obstructive pulmonary disease (COPD), so our results may not be applicable to that particular subset of patients.

## 5 CONCLUSION

In summary, in this cross‐sectional study of mechanically ventilated patients who were eligible for SBT, rises in CVP independently of Hb concentration, PaO_2_, SpO_2_, duration of MV, length of ICU stays, and SBT process, as well as an underlying disease were associated with extubation/weaning failure. It was shown that age, gender, vital signs (MAP, RR, HR), SOFA, and APACHE scores had no significant correlation with patients' extubation outcome. The success or failure of SBT is based on a combination of objectively measured and clinically assessed parameters. The integration of routine CVP measurement in this algorithm should be investigated further in future studies to establish its potential to predict the chance of an earlier diagnosis of patients who would have a failed weaning after SBT. Furthermore, despite limitations, these findings may enable clinicians to execute more timely and effective interventions to maximize the chance of weaning success.

## AUTHOR CONTRIBUTIONS

**Ali Akbar Ghamari**: Conceptualization; data curation; investigation; methodology. **Keivan Amini**: Conceptualization; data curation; investigation; methodology. **Amin Daei Sorkhabi**: Conceptualization; data curation; investigation; methodology; writing—original draft; writing—review & editing. **Aila Sarkesh**: Investigation; writing—original draft; writing—review & editing. **Seyed Hadi Saghaleini**: Investigation; writing—review & editing. **Roghayeh Asghari**: Investigation. **Mansour Rezayi**: Conceptualization; writing—review & editing. **Ata Mahmoodpoor**: Supervision; writing—review & editing.

## CONFLICT OF INTEREST STATEMENT

The authors declare no conflict of interest.

## ETHICS STATEMENT

All procedures were carried out with the approval of the Ethics Committee of Tabriz University of Medical Sciences (IR.TBZMED.REC.1399.364).

## TRANSPARENCY STATEMENT

The lead author Ata Mahmoodpoor affirms that this manuscript is an honest, accurate, and transparent account of the study being reported; that no important aspects of the study have been omitted; and that any discrepancies from the study as planned (and, if relevant, registered) have been explained.

## Data Availability

The data sets used and/or analyzed during the present study are available from the corresponding author on reasonable request.
